# Asymmetrical pedicle subtraction osteotomy for progressive kyphoscoliosis caused by a pediatric Chance fracture: a case report

**DOI:** 10.1186/s13013-017-0115-1

**Published:** 2017-03-14

**Authors:** Satoshi Suzuki, Nobuyuki Fujita, Tomohiro Hikata, Akio Iwanami, Ken Ishii, Masaya Nakamura, Morio Matsumoto, Kota Watanabe

**Affiliations:** Department of Orthopaedic Surgery, Keio University School of Medicine, 35 Shinanomachi, Shinjyuku, Tokyo, 160-8582 Japan

**Keywords:** Chance fracture, Flexion-distraction injury, Kyphoscoliosis, Asymmetrical pedicle subtraction osteotomy, Case report

## Abstract

**Background:**

Although most pediatric Chance fractures (PCFs) can be treated successfully with casting and bracing, some PCFs cause progressive spinal deformities requiring surgical treatment. There are only few reports of asymmetrical osteotomy for PCF-associated spinal deformities.

**Case presentation:**

We here report a case of a 10-year-old girl who suffered an L2 Chance fracture from an asymmetrical flexion-distraction force, accompanied by abdominal injuries. She was treated conservatively with a soft brace. However, a progressive spinal deformity became evident, and 10 months after the injury, examination showed segmental kyphoscoliosis with a Cobb angle of 36°, a kyphosis angle of 31°, and a coronal imbalance of 30 mm. Both the coronal and sagittal deformities were successfully corrected by asymmetrical pedicle subtraction osteotomy.

**Conclusions:**

Initial kyphosis and posterior ligament complex should be evaluated at some point when treating PCFs. Asymmetrical pedicle subtraction osteotomy can be a useful surgical option when treating rigid kyphoscoliosis associated with a PCF.

## Background

Chance fractures, which are flexion-distraction injuries of the spine, were defined by George Quentin Chance in 1948 as a fracture line passing transversely through the spinous process, laminae, and pedicle and then into the vertebral body [1]. Chance fractures account for 5–11% of the acute thoracolumbar spinal injuries in adults [2, 3]. Although pediatric spinal injuries are more unusual, affecting only 0.3–4% of the pediatric population, they have become more common due to mandatory seat-belt laws [4–6]. Of these injuries, about 43–50% consist of pediatric Chance fractures (PCFs) [5]. The treatment of these fractures depends on the fracture pattern as well as neurologic status [5]. Purely osseous injuries with minimal deformity, and even those involving ligamentous injuries, have been treated conservatively, and these injuries have a good prognosis in pediatric patients [7]. However, in some cases, surgery is required to correct a kyphotic deformity or to halt neurological deterioration or a progressive deformity [5, 7]. Surgeries to treat chronic, rigid deformities caused by PCF are rare [8–10]. We here report a case of a 10-year-old girl with rigid, chronic-phase kyphoscoliosis caused by a PCF. The kyphoscoliosis was successfully treated by asymmetrical pedicle subtraction osteotomy (PSO) at the affected vertebra.

## Case presentation

**Fig. 1:**
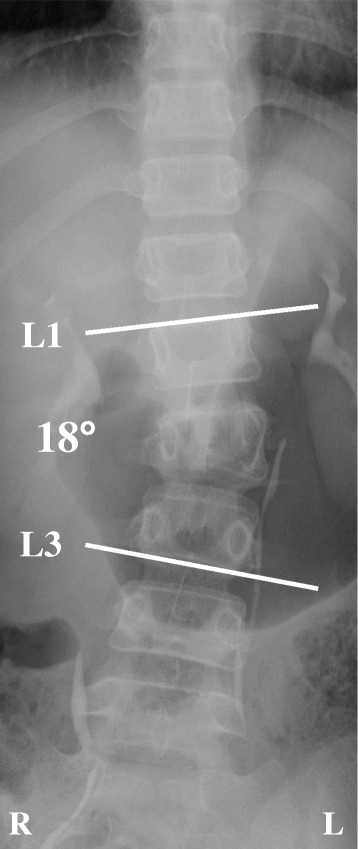
Radiographs at the time of injury. Radiographs obtained immediately after the injury revealed an L2 fracture with local lumbar scoliosis at L1–L3 of 18° (AP view)

**Fig. 2:**
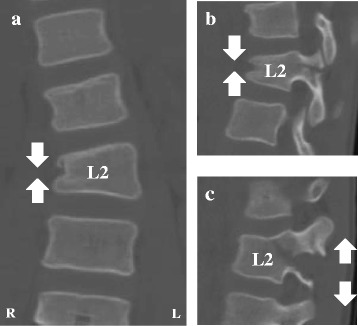
CT images at the time of injury. CT images revealed a Chance-type injury with an associated L2 compression fracture of the right vertebral body (**a**), a horizontal split of the right L2 pedicle (**b**), and the splitting and distraction of the left L2 transverse processes, left L2 pedicle, and the L2 middle column (**c**), resulting in asymmetrical kyphoscoliosis

**Fig. 3:**
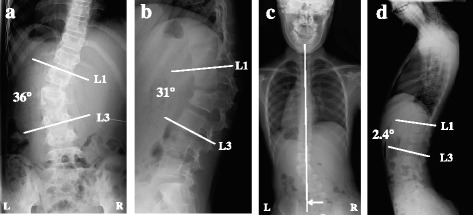
Radiographs at the time of surgery. Lumbar radiographs revealed segmental kyphoscoliosis with a Cobb angle of 36° (**a**) and a kyphosis angle of 31° (**b**). A standing AP view of the entire spine showed a 30-mm leftward shift of the C7-central sacral vertical line (**c**). The kyphotic angle decreased to 2.4° on a lateral radiograph over a bolster (**d**)

**Fig. 4:**
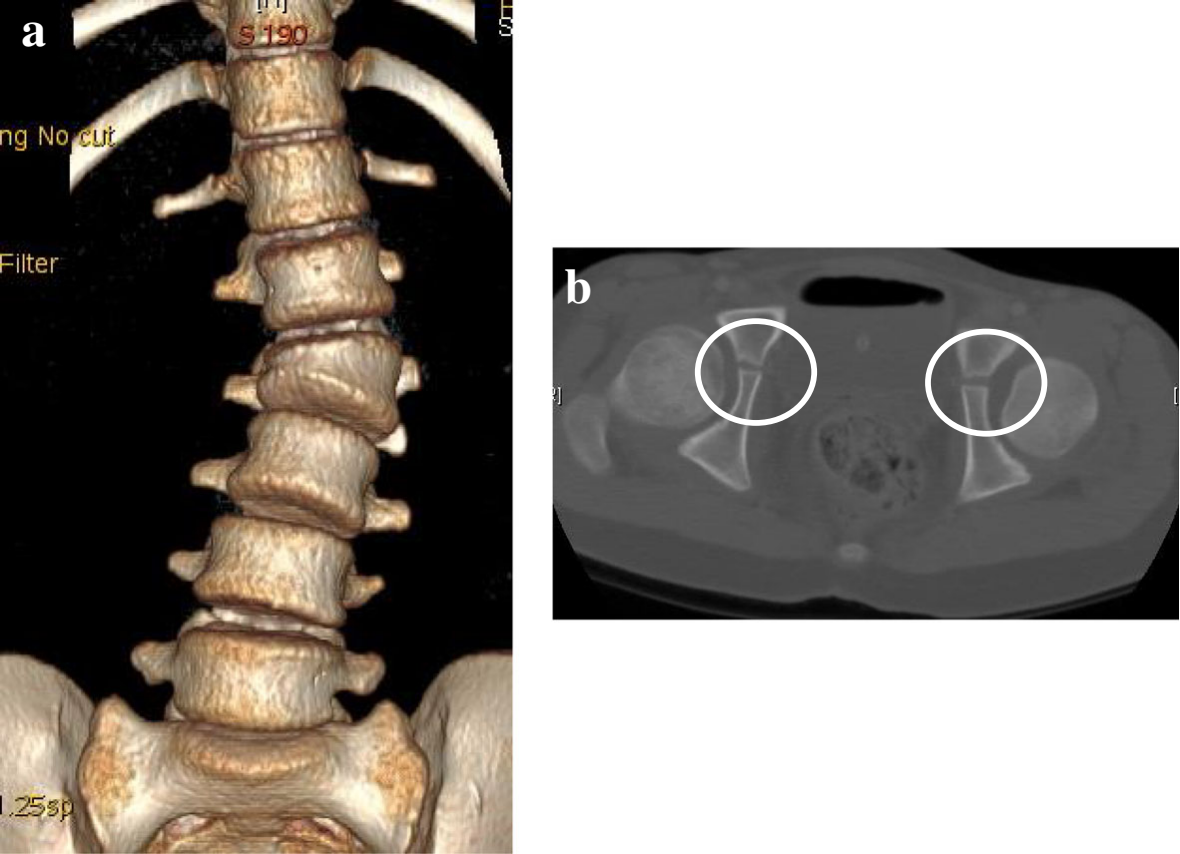
CT images at the time of surgery. A reconstructed three-dimensional CT image showing kyphoscoliosis due to the affected L2 vertebra (**a**). CT images revealed an opening of bilateral Y-shaped cartilage (**b**)

A 10-year-old girl was traveling in the back seat of a car, wearing a 3-point restraint, when the car was involved in a collision at an intersection. The girl was transported to a nearby hospital, where she complained of abdominal and back pain. After clinical and radiographic examination, the diagnoses were perforations in the duodenal and transverse colon, a fracture of the right wrist, and an L2 Chance fracture without neurologic deficit. Radiographs of the lumbar spine revealed local lumbar scoliosis at L1–L3 with a Cobb angle of 18° in a supine position (Fig. 1). CT images showed a horizontal split in the right L2 pedicle (Fig. 2b), a collapse of the right anterior vertebral column (Fig. 2a, b), and splitting of the L2 left transverse processes, the left L2 pedicle, and the middle of the L2 vertebral column (Fig. 2c), resulting in asymmetrical kyphoscoliosis. The girl underwent emergency surgery for the abdominal injury and wrist fracture. The Chance fracture was treated conservatively with 4 weeks of bed rest, after which the patient was allowed to walk with a soft brace. However, the deformity gradually deteriorated, and the girl was referred to our hospital. Physical examination showed that her trunk was leaning to the left side, and standing whole spine radiographs revealed kyphoscoliosis at the thoracolumbar area with a Cobb angle of 36° at L1–L3 (Fig. 3a), a kyphosis angle of 31° at L1–L3 (Fig. 3b), and a coronal imbalance of 30 mm (the distance between the C7 plumb line and the center of the sacrum) to the left (Fig. 3c). The kyphotic angle decreased to 2.4° over a bolster (Fig. 3d). MR images did not show any soft tissue injury or spinal cord damage when she was transferred to our hospital (data not shown). CT images revealed that the fractures had fused (Fig. 4a).

**Fig. 5:**
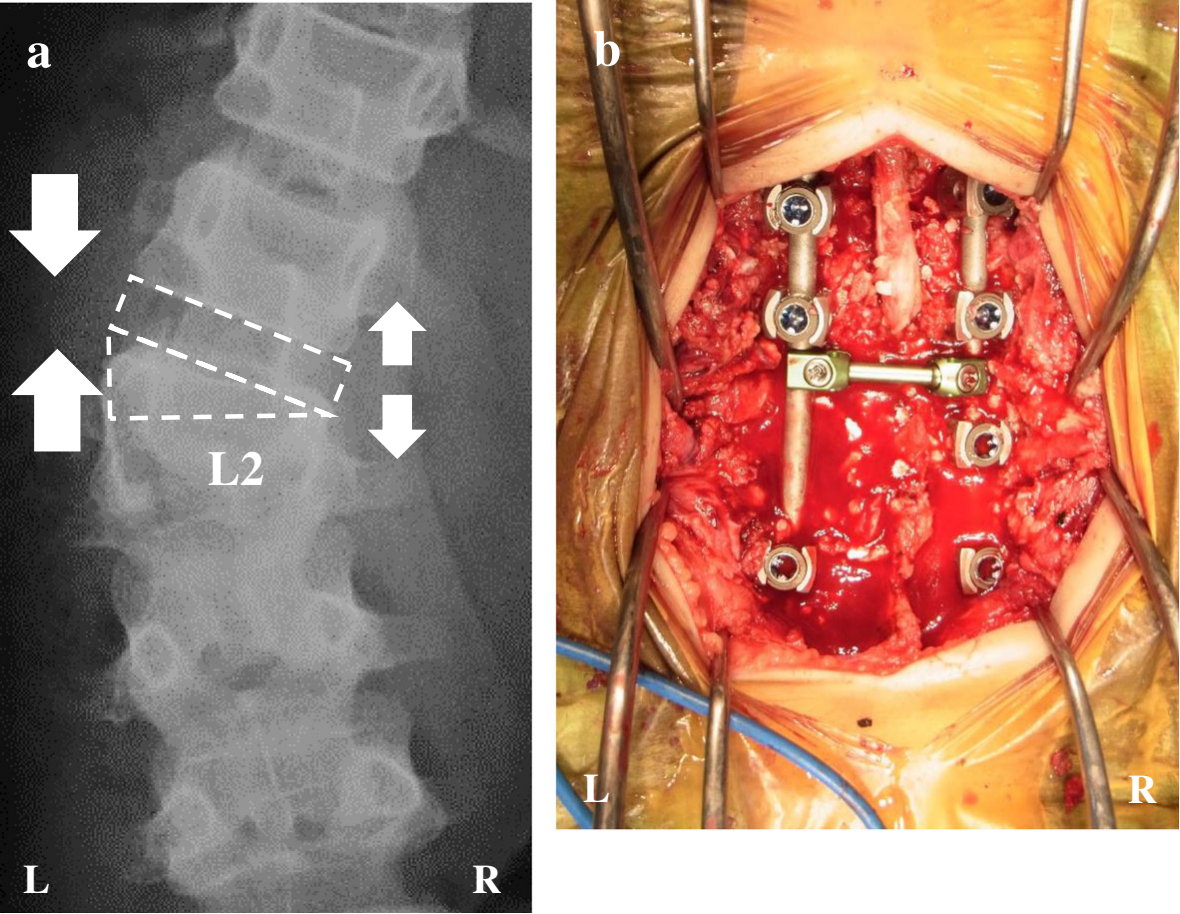
Intraoperative findings. The deformity was corrected by L1/L2 intervertebral disc resection and osteotomy of the upper one third of the elongated L2 pedicle and vertebral body, followed by compression to the left side and distraction to the right side (**a**). An intraoperative photograph just after the correction is shown in (**b**)

Ten months after the injury, we performed correction and fusion surgery using asymmetrical PSO at L2. After exposing the posterior elements through a posterior midline approach, we placed pedicle screws bilaterally at T12, L1, and L3 and at the right L2 pedicle. We next removed the upper one third of the elongated left L2 pedicle, the left upper and posterior portion of the vertebra, and the L1/L2 intervertebral disc (Fig. 5a). After local bone graft into L1/L2 disc space, we applied compression force between the L1 and L3 pedicle screws on the left side while monitoring motor-evoked potentials (Fig. 5a). The facet joints of T12/L1 and L2/L3 and transverse processes of T12, L1, L2, and L3 were decorticated. The remaining local bone graft was placed along the decorticated bones. The intraoperative time was 91 min; the estimated blood loss was 220 ml (Fig. 5b).

**Fig. 6:**
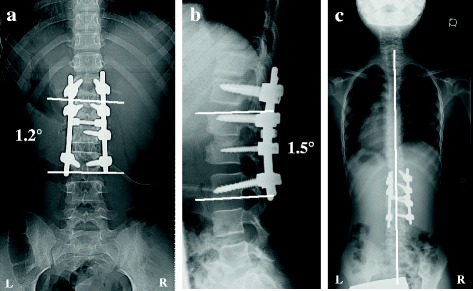
Postoperative radiographs and CT images. Postoperative radiographs revealed that the L1–L3 scoliosis was corrected to 1.2° (**a**) and the kyphosis to 1.5° (**b**). A radiograph and CT image obtained at the 2-year follow-up showed good global coronal balance with no instrument failure or loss of correction (**c**)

The scoliosis was corrected from 36° to 1° and the kyphosis from 31° to 1° (Fig. 6a, b). At the 2-year follow-up, radiographs showed excellent coronal balance, no instrument failure or loss of correction, and osseous continuity between the vertebrae (Fig. 6c).

## Discussion

In 1948, George Quentin Chance characterized a type of fracture with “horizontal splitting of the spine and neural arch [1].” Nicoll proposed the term “Chance fracture” in 1949 [11]. Most cases are associated with a flexion-distraction injury obtained in a motor vehicle accident [12]. In children, however, 2-point restraints and improperly used restraints have increased the incidence of PCFs at the lumbar spine, often at L2 or L3 [7]. As a result of anatomical characteristics, Chance injuries are more likely to cause neurological issues in children than in adults: 15–43% of PCFs involve neurological deficits [7, 13, 14].

### PCF post-traumatic deformities

Delayed displacement and progressive deformities have been reported after conservative therapy for PCF [7, 8, 13, 15, 16]. To summarize the reports, in Chance fractures, the threshold of kyphosis angle for surgical indication was ranged from 15° to 22°. However, taking standing radiographs for assessing initial kyphosis will be difficult in the situation of an acute spinal injury. Additionally, more attention will be paid for the possible concomitant injuries including abdominal viscera and vascular injuries at the time of injury. In our patient’s case, the initial lumbar scoliosis at L1–L3 was 18° in a supine position. Based only on the scoliosis, conservative treatment was a reasonable choice. Additionally, since treatments for abdominal injuries had the priority in this case, the delay in the evaluations for kyphosis angle and damages of posterior ligament complex were inevitable. The case report is therefore an important lesson of what can happen if an unstable asymmetrical Chance fracture is not well managed in the acute phase after trauma.

### Treatment of PCFs

Outcomes of conservative therapy for PCFs are relatively good; however, some PCFs should be treated surgically to correct an initial kyphotic deformity or to prevent further neurological deterioration or a progressive deformity [17]. Since the injury of PCFs is mainly the posterior osteoligamentous complex, reduction and stabilization with posterior instrumentation should be considered [18]. When treating acute PCFs surgically, pedicle screw instrumentation, which extended one or two levels above and below the affected vertebra, seems to be a popular treatment [19]. With a recent progress of spinal instrumentations, percutaneous pedicle screw fixation may have evolved as an alternative approach for PCFs [5, 20]. On the other hand, there are only a few reports of surgical treatment for chronic deformities due to PCFs, including combined anterior and posterior fusion surgery, transforaminal thoracic interbody fusion, and transpedicle wedge osteotomy and posterior fusion [8, 21, 22]. Asymmetrical PSO, which was first reported in 2012 by Sathya et al. [23], is not a novel technique; however, it seems to be rare rerated to a report of asymmetrical PSO for a chronic pediatric Chance fracture. Our patient’s spinal deformity was caused by a deformity of the fractured L2 vertebra, so we judged that short fusion with asymmetrical PSO was sufficient to correct the affected vertebra. Our osteotomy procedure included partial resection of the pedicle, vertebral body, and adjacent disc in an applied grade 4 osteotomy, according to the classification system of anatomically based spinal osteotomies proposed by Schwab et al. [24]. In this case, we intended to fuse from T12 to L3 for the maintenance of spinal alignment after correction of scoliosis and kyphosis. The application of without fusion technique at L2/L3 fact joints and future removal of the spinal implants might be another option to preserve motion segment at L2/L3. However, we have removed posterior ligamentous complex at L2/L3 during the surgery and were afraid of the occurrence of distal junctional problem after removal of the implants.

## Conclusions

Initial kyphosis and posterior ligament complex should be evaluated at some point when treating PCFs. Asymmetrical PSO is not a novel technique; however, there are only few reports of asymmetrical osteotomy for PCF-associated spinal deformities. Asymmetrical pedicle subtraction osteotomy can be a useful surgical option when treating rigid kyphoscoliosis associated with a PCF.

## Abbreviations

CT: Computed tomography
MR: Magnetic resonance
PCF: Pediatric Chance fracture
PSO: Pedicle subtraction osteotomy
